# Golden-step phase encoding for flexible realtime Cardiac MRI

**DOI:** 10.1186/1532-429X-13-S1-P23

**Published:** 2011-02-02

**Authors:** John A Derbyshire, Haris Saybasili, Liheng Guo, Ozan Sayin, Peter Kellman, Robert J Lederman, Daniel A Herzka

**Affiliations:** 1 NHLBI, National Institutes of Health, DHHS, Bethesda, MD, USA; 2 Johns Hopkins School of Medicine, Baltimore, MD, USA

## Introduction

Realtime MRI involves an inevitable trade-off between spatial resolution, temporal resolution, field-of-view (FOV), and signal-to-noise (SNR) ratio. In conventional Cartesian imaging all these parameters are fixed prior to scanning, and images can typically only be reconstructed for the prescribed parameters.

Previously, Winkelmann [1] demonstrated the Golden Angle radial technique, advancing the projection angle in 111° steps (the Golden Ratio of 180°), providing almost uniformly distributed projections after any arbitrary number of acquisitions.

Here we propose and demonstrate the use of Golden Step phase encoding for Cartesian type acquisitions permitting the temporal resolution, FOV and SNR to be selected retrospectively and multiple reconstructions with varied parameters from the same data.

Applications include interventional imaging where images are required to serve multiple purposes simultaneously (e.g. instrument guidance and subject monitoring).

## Methods

**Figure 1:**
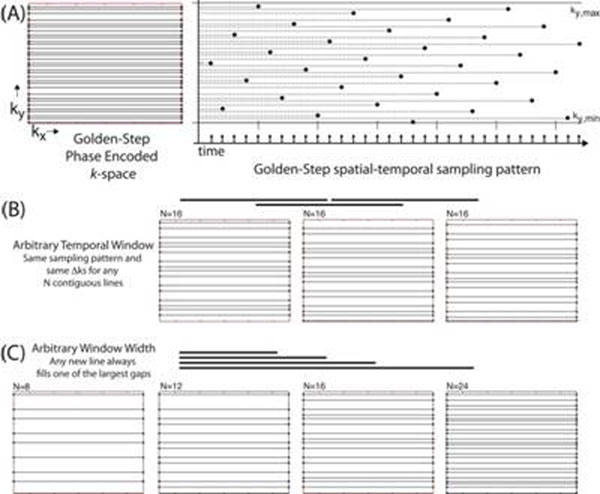
Golden step phase encoding k-space filling strategy

Two normal volunteers were imaged with prior, written informed consent and local IRB approval. MRI was performed using a 1.5T Avanto system (Siemens, Erlangen, Germany), a 32-channel cardiac array coil (Rapid Biomedical, Rimpar, Germany) and RF-spoiled GRE and balanced SSFP sequences, modified for Golden Step by advancing the phase encoding by Golden Fraction (0.6180339) of the k-space support region at each TR (see Figure 1).

Breath-hold and free-breathing ungated, continuous imaging was performed in cardiac short- and long-axis slices over 20s intervals. Imaging with a 128 sample, 488Hz/pixel readout and TE/TR=2.12/4.26ms provided ~4500 TRs. and 2.5x2.5x7mm resolution.

Images were reconstructed in MATLAB (The MathWorks, Natick, MA) by direct linear inversion (pinv()) of the MR image encoding process for each coil and golden phase encode step.

## Results

**Figure 2:**
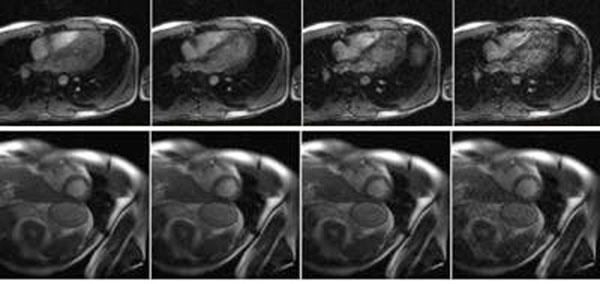
Long and short axis golden step cardiac images with R=1-4

Example images reconstructed for differing temporal resolutions and acceleration rates R=1-4 from a single acquisition are shown in Figure 2.

## Conclusions

Golden step phase encoding allows imaging multiple simultaneous temporal resolutions and permits the retrospective selection of acceleration rate.

Golden step imaging yields close-to-uniformly sampled k-space for any number of TRs permitting arbitrary selection of both the temporal window width and position. These data are suitable for self-calibrated parallel reconstruction with implicit selection of acceleration rate.

As expected, image noise increases with increasing temporal resolution (less acquired data and higher parallel imaging acceleration). In comparison to equivalent standard Cartesian imaging, noise is very marginally increased due to slightly non-uniform sampling of k-space.
